# Complete genome sequences of *Vibrio parahaemolyticus* strains L2171 and L2181 associated with AHPND in *Penaeus vannamei* postlarvae by hybrid sequencing

**DOI:** 10.1016/j.dib.2025.111819

**Published:** 2025-06-22

**Authors:** Guillermo Reyes, Betsy Andrade, Irma Betancourt, Bonny Bayot

**Affiliations:** a Centro Nacional de Acuicultura e Investigaciones Marinas, CENAIM, Escuela Superior Politécnica del Litoral, ESPOL, Guayaquil, Ecuador; b Facultad de Ingeniería Marítima y Ciencias del Mar, FIMCM, Escuela Superior Politécnica del Litoral, ESPOL, Guayaquil, Ecuador

**Keywords:** Pathogenic bacteria, Pacific white shrimp, Whole-genome sequencing, PirAB toxin genes, Illumina NovaSeq, PacBio Revio, Virulence factors

## Abstract

*Vibrio parahaemolyticus* strains L2171 and L2181 were isolated from a *Penaeus vannamei* shrimp hatchery. Both strains carry the pVA plasmid harboring the *PirAB* genes encoding the binary PirAB toxins that cause the acute hepatopancreatic necrosis disease (AHPND) in cultured shrimp. The strains also harbor multidrug resistance (MDR) and a repertoire of virulence factor genes. Our goal was to determine their complete genome sequences and perform a comprehensive analysis of their genetic characteristics. Therefore, the genomes of two strains, which are highly virulent to shrimp were sequenced by Illumina and the PacBio platforms. These data contribute to a better understanding of *V. parahaemolyticus* and its role as a pathogen in commercially important species such as farmed shrimp, providing valuable insights for disease management in aquaculture.

Specifications Table

**Table:** 

Subject	Omics: Genomics
Specific subject area	Pathogenic genome: Genomics, Bioinformatics, and Phylogeny.
Type of data	Figures: Circular map of *Vibrio parahaemolyticus* genomes, phylogenetic tree analysis. FASTA: Genomic sequence data.
Data collection	Two bacterial strains were isolated from *P.vannamei* postlarvae sampled from tanks during a mortality event and with clear signs of infection by AHPND-causing bacteria. Genomic DNA: Quick-DNA HMW MagBead Kit (Zymo Research, USA); DNA quality check: Dnovix DS-11 Spectrophotometer (Dnovix, USA) and 2 % (w/v) agarose horizontal gel electrophoresis; Sequencing Illumina NovaSeq X plus and PacBio Revio; Assembly: NextDenovo; genome identification and annotation: PGAP, NCBI; Percentage identity: BLAST nucleotide, NCBI; Multiple sequence alignment and Phylogenetic tree: TYGS.
Data source location	The bacteria were isolated from postlarvae 7 in commercial hatchery tanks in South America.
Data accessibility	Repository name: These genomes of *Vibrio parahaemolyticus* have been deposited in GenBank. Data identification number: Whole genome assembly and annotation of *Vibrio parahaemolyticus* L2171 (CP176031 to CP176034) and L2181 (CP176035 to CP176037) Direct URL to data: https://www.ncbi.nlm.nih.gov/nuccore/CP176031 https://www.ncbi.nlm.nih.gov/nuccore/CP176032 https://www.ncbi.nlm.nih.gov/nuccore/CP176033 https://www.ncbi.nlm.nih.gov/nuccore/CP176034 https://www.ncbi.nlm.nih.gov/nuccore/CP176035 https://www.ncbi.nlm.nih.gov/nuccore/CP176036 https://www.ncbi.nlm.nih.gov/nuccore/CP176037
Related research article	None.

## 1 Value of the Data

• These data provide the sequence of *Vibrio parahaemolyticus* genomes, which will be valuable for the molecular taxonomy and phylogenetics of AHPND-causing bacteria in *Penaeus vannamei* hatcheries, as well as for monitoring.
• This genomic data will allow researchers to assess the level of virulence to the genetic variation encoded in the *PirA* and *PirB* plasmids.
• The complete genomes offer valuable insights for understanding the virulence mechanisms of *Vibrio parahaemolyticus* in shrimp hatcheries*.*

## 2 Background

*V. parahaemolyticus* is a ubiquitous marine bacterium and one of the most common causes of shrimp mortality in aquaculture worldwide [1]. Certain strains of this bacterium are responsible for shrimp diseases collectively referred to as vibriosis, including acute hepatopancreatic necrosis disease (AHPND), which has been reported since 2016, particularly in *Penaeus vannamei* juveniles [2]. The pathogenicity of AHPND-causing strains is primarily attributed to the presence of the *pVA* plasmid, which encodes two homologous toxin genes, *PirA* and *PirB* (*PirAB*) [3,4]. While considerable research has focused on understanding AHPND and vibriosis in juvenile shrimp [5,6], there is limited information on the genetic characterization of *V. parahaemolyticus* strains affecting larval stages. This gap underscores the critical need for comprehensive genomic information on *V. parahaemolyticus* strains, as it can provide essential insights for the development of effective strategies to control and manage AHPND in shrimp hatcheries.

## 3 Data Description

We present the complete genomes of *V. parahaemolyticus* strains L2171 and L2181 isolated from shrimp hatchery tanks during a mortality event and sequenced using the Illumina NovaSeq X Plus and PacBio Revio platforms. The genomes of strains L2171 and L2181 are 5,407,932 bp and 5,310,877 bp in length, respectively, and are organized into two chromosomes. Strain L2171 contains 4,986 genes, while strain L2181 has 4,882 genes. Both strains harbor the plasmid pVA, which encodes the *PirAB* toxin genes. Additionally, strain L2171 contains an unidentified plasmid. Both strains feature two CRISPR arrays and exhibit a high abundance of COGs associated with transcription, signal transduction, energy production, ribosomal biogenesis, and cell wall biogenesis. These genetic traits highlight their potential for enhanced environmental adaptability, energy efficiency, and cellular resilience.

The *V. parahaemolyticus* L2171 genome contains several genes encoding enzymes involved in sporulation, sulfide metabolism, viral defense response, and bacteriocin transport. Both strains carry two CRISPR arrays each and 12 antimicrobial resistance (AMR) genes, including *van (T, C, G), CRP, tet* (35, 59), *txR, sul2, rsmA, adeF, CARB-18*, and *parE*, which are associated with resistance to vancomycin, erythromycin, tetracycline, sulfadiazine, chloramphenicol, and others. A total of 159 virulence factor genes were detected across the genomes (*Mannose-Sensitive Hemagglutinin, Type III Secretion System 1, Type III Secretion System 2, Exopolysaccharides*, among others), with the *pVA* plasmid, carrying *PirAB* toxin genes, present in both strains. High COG category abundances were observed in transcription, signal transduction, energy production, and ribosomal and cell wall biogenesis processes.

## 4 Experimental Design, Materials, and Methods

Two bacterial strains were isolated from macerated *P. vannamei* postlarvae 7 sampled from tanks during a mortality event and with clear signs of infection by AHPND-causing bacteria. The sample was cultured on TCBS agar, with strains purified on TSA plates and selected based on morphology and abundance. High molecular weight DNA was extracted using the Quick-DNA HMW MagBead Kit (Zymo Research, USA), and its quality and concentration were assessed using a DeNovix DS-11 spectrophotometer (DeNovix Inc., USA) and agarose gel electrophoresis. Whole genome library preparation and sequencing was performed by Novogene Inc. (Sacramento, USA) using the Illumina NovaSeq X Plus and PacBio Revio platforms.

Whole-genome assembly from long-read sequences was performed using NextDenovo v2.5.2 [7], yielding two chromosomes for each strain and one plasmid, designated pVA (Table 1). Notably, an additional unidentified plasmid was assembled in strain L2171. Assemblies were subsequently polished using NextPolish2 v0.2.1 [8], integrating both PacBio HiFi reads, and short reads generated by the Illumina NovaSeq X Plus platform. Initially, HiFi reads were mapped to the assemblies using Winnowmap [9], with repetitive regions identified by constructing a 15-mer database through Meryl v1.4.1 [10]. Short reads were quality filtered using Fastp v0.23.4 [11], followed by the generation of 21-mer and 31-mer datasets using Yak v0.1 . The resulting HiFi mapping data and k-mer datasets were used as input for the polishing process.

**Table 1:** Assembly details and annotated genome features of the two *Vibrio parahaemolyticus* strains L2171 and L2181.

Item	Value	
	**L2171**	**L2181**
Genome length (bp)	5,407,932	5,310,877
Chromosomes	2	2
Coverage (X)	571	877
GC content (%)	45.24	45.29
Total of genes	4,986	4,882
Total No. of CDS	4,720	4,632
rRNAs	13, 12, 12 (5S, 16S, 23S)	13, 12, 12 (5S, 16S, 23S)
tRNAs	133	133
ncRNAs	4	4
Pseudogenes	92	76
Plasmids	2 (pVA and unidentified plasmid)	1 (pVA)
Assembly accession number	CP176034	CP176037

**Fig. 1:**
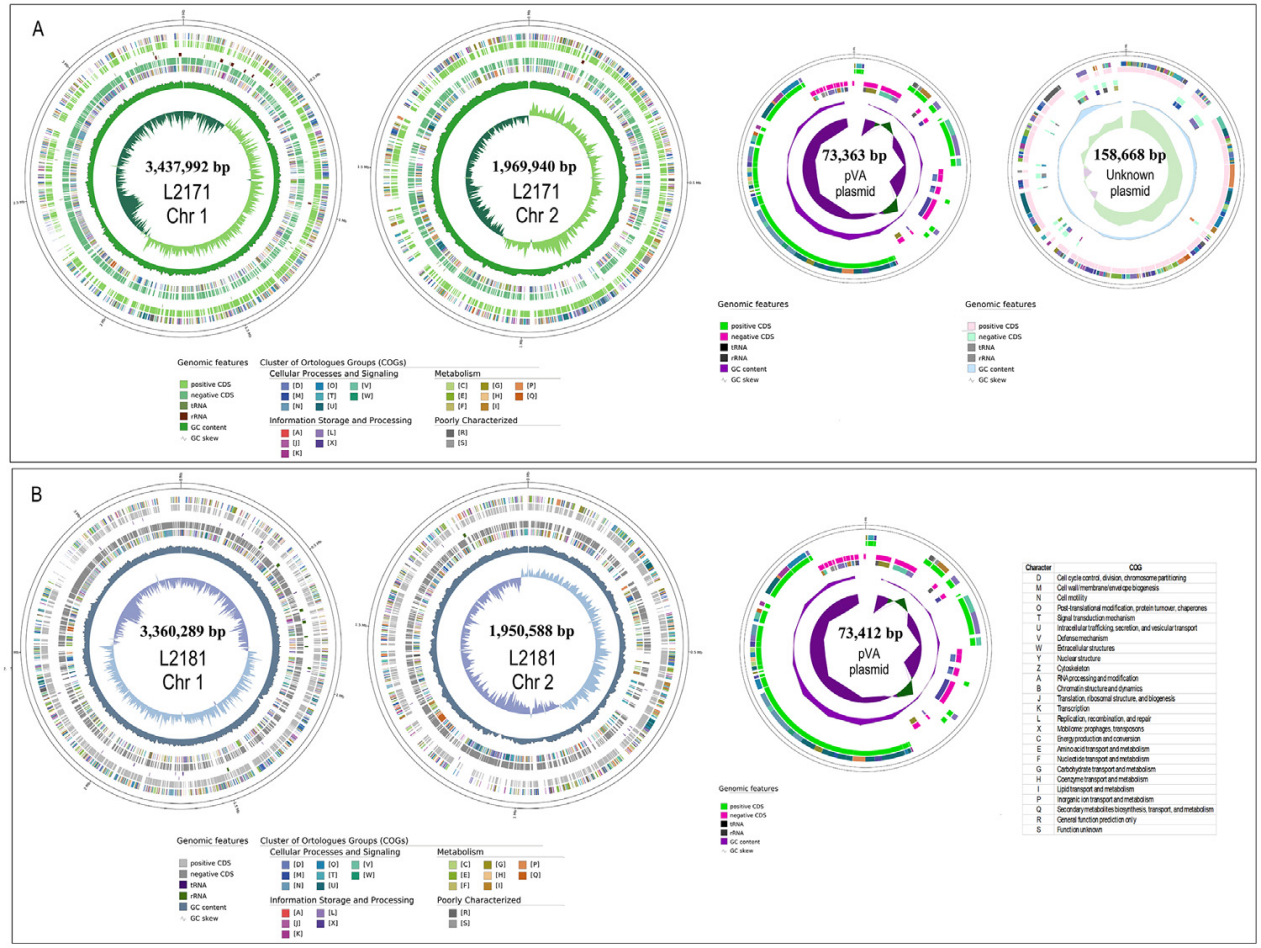
Genome representation of *Vibrio parahaemolyticus* L2171 and L2181. (A) Genome map for L2171: circles from inside to outside: GC skew, GC content, rRNA, tRNA, negative CDS, and positive CDS. (B) Genome map for L2181. The functional categories of genes are annotated based on the COG database. The accompanying table provides a detailed explanation of the colour codes.

Genome quality was assessed using QUAST v5.3.0 [12], and annotation was performed with the NCBI Prokaryotic Genome Annotation Pipeline (PGAP) [13]. A circular genome map (Fig. 1) was generated using GenoVi [14]. Taxonomic classification of strains L2171 and L2181 identified them as *V. parahaemolyticus*, based on Kraken v2.0 and 16S rRNA gene sequence homology analysis via BLAST. Average nucleotide identity (ANI) analysis with fastANI v1.34 [15] showed identity values of 99.62 % and 99.38 % for the L2171 and L2181 strains, respectively, compared to the *V. parahaemolyticus* reference genome (RIMD 2210633, GenBank GCA_008632335.1). Antimicrobial resistance (AMR) genes were identified using the Resistance Gene Identifier (RGI) v6.0.3 [16], and CRISPR arrays were detected with CRISPRCasFinder v4.2.20 [17,18]. In addition, the virulence factors of both strains were characterized using the Virulence Factor Database (VFDB) [19].

**Fig. 2:**
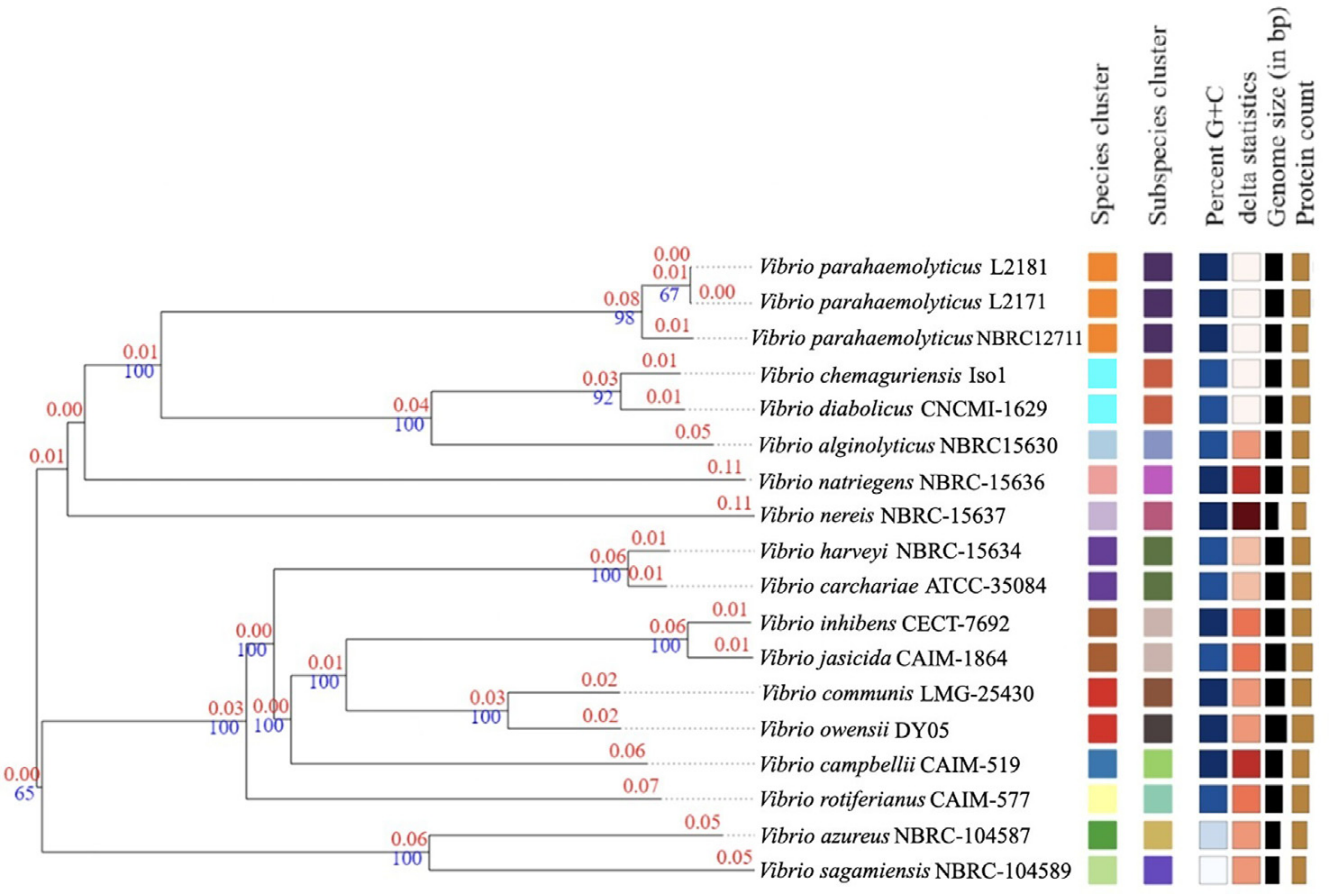
Phylogenetic tree of *Vibrio* genomes, including *V. parahaemolyticus* L2171 and L2181, along with related species, revealed that two strains clustered within the orange group. This cluster comprised species and subspecies of *Vibrio parahaemolyticus that shared* a similar G+C content percentage. Red numbers above branches represent evolutionary distances, while blue numbers at branching points indicate bootstrap support (%).

A genome-based phylogenomic analysis was performed (Fig. 2) using TYGS [20]. Nomenclature information was obtained from LPSN (available at https://lpsn.dsmz.de) [21]. The genomes of *V. parahaemolyticus* L2171 and L2181 were aligned with the genomes of all type strains available in the TYGS database using the MASH algorithm [22], and the ten strains with the smallest MASH distances were selected and calculated using the GBDP method.

## Limitations

Not applicable.

## Ethics Statement

This study involved work with bacterial strains and did not include experiments with humans or animals. The bacterial strains were handled by institutional guidelines and standard microbiological safety practices. Therefore, no ethical approval was required for this work.

## CRediT authorship contribution statement

**Guillermo Reyes:** Formal analysis, Methodology, Investigation, Data curation, Writing – original draft, Visualization, Software, Writing – review & editing. **Betsy Andrade:** Conceptualization, Methodology, Writing – review & editing. **Irma Betancourt:** Conceptualization, Methodology, Writing – review & editing. **Bonny Bayot:** Supervision, Funding acquisition, Writing – review & editing.

## Data Availability

(NCBI).Whole genome assembly and annotation of Vibrio parahaemolyticus (Original data)
